# Integrated human-virus metabolic stoichiometric modelling predicts host-based antiviral targets against Chikungunya, Dengue and Zika viruses

**DOI:** 10.1098/rsif.2018.0125

**Published:** 2018-09-12

**Authors:** Sean Aller, Andrew Scott, Mitali Sarkar-Tyson, Orkun S. Soyer

**Affiliations:** 1 School of Life Sciences, University of Warwick, Gibbet Hill Campus, Coventry CV4 7ES, UK; 2 Defence Science and Technology Laboratory (Dstl), Porton Down, Salisbury SP4 0JQ, UK; 3 Marshall Center for Infectious Disease Research and Training, School of Biomedical Sciences, University of Western Australia, Perth, Australia

**Keywords:** emerging viruses, antiviral targets, flux balance analysis, host–virus interactions, metabolic modelling

## Abstract

Current and reoccurring viral epidemic outbreaks such as those caused by the Zika virus illustrate the need for rapid development of antivirals. Such development would be facilitated by computational approaches that can provide experimentally testable predictions for possible antiviral strategies. To this end, we focus here on the fact that viruses are directly dependent on their host metabolism for reproduction. We develop a stoichiometric, genome-scale metabolic model that integrates human macrophage cell metabolism with the biochemical demands arising from virus production and use it to determine the virus impact on host metabolism and vice versa. While this approach applies to any host–virus pair, we first apply it to currently epidemic viruses Chikungunya, Dengue and Zika in this study. We find that each of these viruses causes specific alterations in the host metabolic flux towards fulfilling their biochemical demands as predicted by their genome and capsid structure. Subsequent analysis of this integrated model allows us to predict a set of host reactions, which, when constrained, inhibit virus production. We show that this prediction recovers known targets of existing antiviral drugs, specifically those targeting nucleotide production, while highlighting a set of hitherto unexplored reactions involving both amino acid and nucleotide metabolic pathways, with either broad or virus-specific antiviral potential. Thus, this computational approach allows rapid generation of experimentally testable hypotheses for novel antiviral targets within a host.

## 1. Introduction

The rapid development of antiviral drugs for emerging and re-emerging viruses, such as the Zika virus, remains a significant challenge [1,2]. Given that virus production within a host is intertwined with host immune response and metabolism [3], it is suggested that new development of antivirals should take into account host processes [4,5]. Indeed, viruses are entirely dependent on their hosts' cellular resources for their replication. This is highlighted by observed variations in virus production levels correlating with cell-to-cell variance in growth rate and phase [6], as well as virus infection leading to changes in host metabolism [7]. In particular, virus infection leads to significant metabolic alterations in the host, in some cases resulting in up to threefold increase in glycolysis rates [7–9] and changes in ATP production rates [6]. This observation can be seen as an emergent property of the combined host–virus metabolic system and could be related to changes in host cellular demands arising from viral production [10,11]. More specifically, alterations in host metabolism upon infection can be understood as viruses actively manipulating the host system to their advantage [12], or the additional draw of metabolic components for viral production simply resulting in a rearrangement of host metabolic fluxes.

Regardless of its cause, the entanglement between host metabolism and viral production opens up the possibility to perturb the former, as a way of limiting the latter [9,12,13]. To explore this possibility and towards understanding the potential interplay between host metabolism and the additional ‘virus demand’ on it, stoichiometric genome-scale metabolic models and their optimization through flux balance analysis (FBA) can provide ideal starting points as they are demonstrated to allow analysis of cellular physiology as an interconnected system [14,15]. Integration of virus production in a host metabolic model has already been used to study the infection of bacteria with phage, indicating the presence of metabolic limitations on phage replication depending on the host's metabolic environment [16]. Such studies have shown that while FBA does not allow for the simulation of a virus infection over time, it provides valuable insights into the metabolic rearrangements that occur from an uninfected to infected state [16]. Theoretically, this type of stoichiometric metabolic analysis can potentially be applied to any host–virus pair; it is particularly suited to *Alpha*- and *Flavi*-viruses. The rather simple physical and genomic structure of these viruses [17,18] allows straightforward construction of a pseudo biochemical reaction representing their production from constituting parts. This pseudoreaction can then subsequently be incorporated into a genome-scale metabolic model of any host.

Here, we develop and apply such an FBA approach to analyse host–virus metabolic entanglement. We focus this analysis on representatives of the virus genera *Alpha-* (CHIKV) and *Flavi-virus* (DENV, ZIKV), of the *Togaviridae* and *Flaviviridae* virus families, which are positive-sense single-strand RNA viruses with rather simple physical structures [17,18]. Viruses of both families have been observed to infect many different human cell types [19,20], including monocyte-derived macrophage cell lines [19,21,22], and are usually transmitted to humans via arthropod vectors, the most common being mosquitoes of the *Aedes* genus [23,24]. By analysing the integrated metabolic model, we find that viral production results in significant alterations in host metabolic fluxes, including changes in central carbon metabolism and lipid biosynthesis pathways. These changes have led us to postulate that a set of host reactions can be constrained in such a way to inhibit virus production. We show that this approach can indeed allow prediction of key virus-limiting host reactions, which overlap with known targets of existing antiviral drugs, such as those targeting host nucleotide pathways. In addition, our predictions highlight a set of hitherto unexplored host reactions as potential antiviral targets.

## 2. Results

To analyse host–virus interaction from a metabolic stance, we developed here an integrated stoichiometric model of a human macrophage cell infected with a virus. This integrated virus–macrophage metabolic modelling approach was considered here for three viruses causing recent epidemic outbreaks: Chikungunya virus (CHIKV); Dengue virus (DENV); and Zika virus (ZIKV). For each virus, we integrated a biomass reaction into the human model that represents the production of virus particles. While the viral genome and protein stoichiometry are available for most species of *Alpha-* and *Flavi-virus* genera, the detailed stoichiometric quantification of their lipid envelopes is mostly lacking [17,18]. Thus, the presented analysis is based only on the amino acid and nucleotide requirements constituting the virus biomass function (see *Methods* and electronic supplementary material, figure S1 for details of virus biomass calculations).

### 2.1. Host metabolism displays alternative host- and viral-optimal states

**Figure 1.**
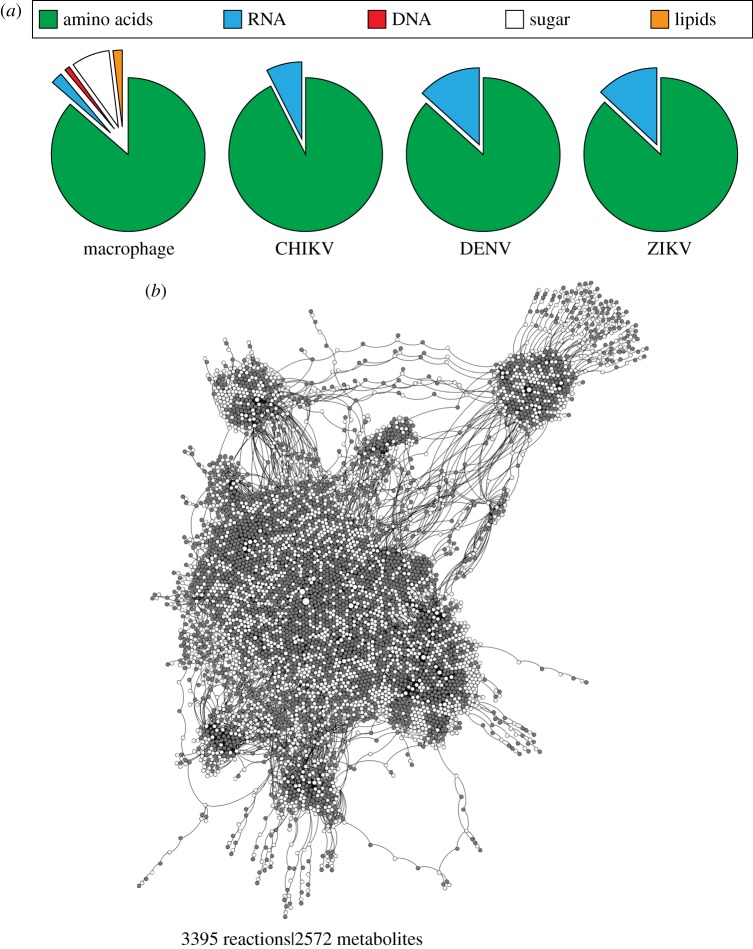
Comparison of host macrophage and viral biomass compositions, and metabolic. (*a*) Comparison of host macrophage, CHIKV, DENV and ZIKV biomass compositions, as described from their respective biomass objective functions, using five different macromolecular classes (amino acids, RNA, DNA, sugar and lipids. Full breakdown of biomass is available in electronic supplementary material, file S1). (*b*) Bipartite graph visualization of the macrophage metabolic network, where nodes are metabolites (white fill) or reactions (grey fill), edges are connections between them and indicate directionality. CHIKV, Chikungunya virus; DENV, Dengue virus; ZIKV, Zika virus.

We first used the integrated virus–macrophage stoichiometric metabolic model to interrogate potential changes in host metabolism upon virus infection. To do so, we considered two idealized scenarios: (i) the metabolic system is optimized for the functional requirements of the host cell as determined by a maintenance-related biomass reaction [25] (host-optimal state); (ii) the metabolic system is optimized solely for virus particle production (virus-optimal state). These two states provide the theoretical extremes of a continuum of metabolic states that can arise during virus infection. While the first scenario aims to represent the normal physiological state of macrophage cells, the second state represents a thought experiment of the host metabolic fluxes being set for maximizing virus production (figure 1*a*,*b*).

**Figure 2.**
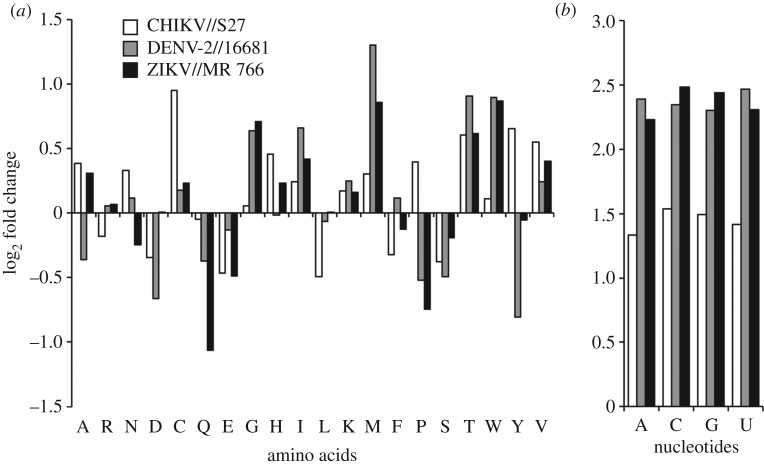
Fold-change difference in usage of amino acids and nucleotides between host and CHIKV, DENV and ZIKV. (*a*,*b*) The usage of amino acids (*a*) and nucleotides (*b*) between the host and virus biomass objective functions. The differential usage was calculated against all biomass precursors. Comparison was conducted for all 20 amino acids, and four RNA nucleotides (the *x*-axes are labelled with the standard short notations for these). All calculations and biomass formulations are as described in the *Methods*, and all biomass stoichiometric values are provided as electronic supplementary material, file S1.

To compare the host- and virus-optimal states of the model, we analyse the metabolic fluxes directly feeding into the biomass pseudoreaction (see electronic supplementary material, files S1 for biomass reactions and S2 for flux values and ranges). This is done by analysing the fluxes that directly produce a given biomass precursor (e.g. alanine) in both the host- and virus-optimized states. As expected from linear optimization, we find that the difference in these fluxes (for the two optimization cases) reflects the stoichiometric differences in the amino acid and nucleotide requirements of the host cell and the virus, thus achieving perfect fulfilment of host or virus biomass requirements. We conclude that stoichiometric differences in metabolic requirements for virus production versus host maintenance, as summarized in figure 2, result in different metabolic flux states of the host model.

### 2.2. Host- and virus-optimal metabolic states require non-overlapping flux ranges in vital metabolic subprocesses

**Figure 3.**
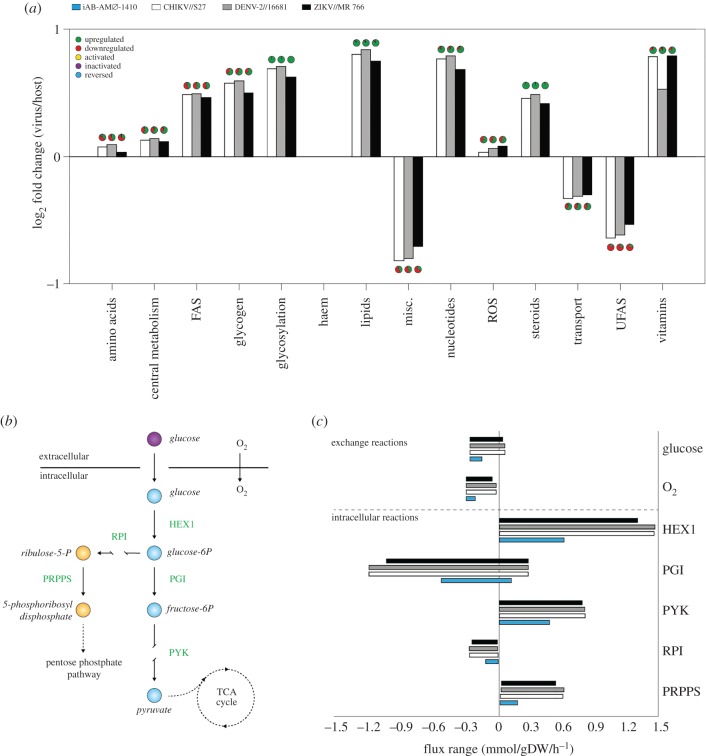
Comparison of model fluxes between host optima and CHIKV, DENV and ZIKV optima. (*a*) Comparisons are visualized as the sum of fluxes over aggregated subsystems using values from host- and virus-optimal states. Abbreviations used in the subsystem classification are: FAS, fatty acid synthesis; ROS, reactive oxygen species; UFAS, unsaturated fatty acid synthesis; Misc., miscellaneous. The *y*-axis represents differential usage of aggregate subsystems, while the colours of the bars indicate different viruses and host (see colour coding on the panel). Positive and negative values reflect a higher or lower total flux for that subsystem in the virus- compared to host-optimal state. Pie charts over each bar provide a summary of changes on individual reactions within a subsystem. The complete set of flux values for all reactions in the model and all optimal states are provided as electronic supplementary material, file S1*.* (*b*) Simplified schematic showing reactions involved in the glycolysis pathway. (*c*) Corresponding flux ranges of individual reactions in the glycolysis pathway that allow attainment of host and virus optima. The flux ranges allowing optima for individual viruses, as well as the host, are shown in differentially coloured bars, with the *x*-axis showing flux values. The colour coding is as shown in panel *a*.

To understand how the flux changes at the biomass level affect the metabolic system, we calculated the allowed flux variability for individual reactions in the model using either host- or virus-based optimization (see *Methods*). Flux variability analysis (FVA) allows for a more robust analysis of different states of the model, compared to merely calculating optimal flux sets, which are shown to be subject to inaccuracies inherent in linear solvers used in FBA [26]. We find that the median of the allowed optimal metabolic flux ranges, between host- and virus-optimal states, shows significant changes across different metabolic processes (called ‘subprocesses’ from now on) (figure 3 and electronic supplementary material, file S2). In particular, the virus-optimal state displays significantly increased median flux for reactions associated with lipid metabolism and nucleotide biosynthesis and significantly decreased flux for reactions associated with unsaturated fatty acid biosynthesis and transport (including intracellular transport reactions). Besides these general overall trends across subprocesses, the virus-optimal state displays also differences in the median flux of specific reactions within each subprocess (see pie charts in figure 3). These changes are per downstream requirements for fulfilling biomass requirements and relate to interconnections among subprocesses. For example, reactions showing the most increase from host- to virus-optimised flux states involve ADP/ATP and inorganic phosphor (Pi), signifying a shift from the lipid-production in the host-optimal state to phosphate-reclaim in the virus-optimal state. These metabolites, specifically phosphate and phosphate derivatives in the latter example, link directly into the reactions of the nucleotide biosynthesis subprocess (which then feed into increased nucleotide requirement in the virus, see figure 2).

The specific change in the allowed flux ranges also highlight potential physiological changes. As an illustrative example, we show the extent of changes within the glycolysis pathway, where allowed flux ranges that can sustain virus optima are wider compared to those that can sustain host optima (figure 3). The allowed ranges for glucose and oxygen uptake indicate that virus optima can be sustained even under low-uptake fluxes, indicative of the potential feasibility of anaerobic metabolism still sustaining virus production [27]. Taken together, this comparison of host- and virus-optimal states show that the differences within the stoichiometric requirements of the different viruses and the host cause large-scale alterations in the host metabolic fluxes.

### 2.3. The model-predicted differences between host- and virus-optimal metabolic states match metabolite-based observations from infected cells

As discussed above, the model predictions up to this point arise from a thought experiment in which we compare fluxes from the host metabolic system optimized for either host maintenance or viral production. While a full shift of host metabolism to supporting viral production is unlikely, this comparison can still provide insights into how metabolic fluxes in a host might shift with virus infection. To see if the model predictions match with biological observations, we attempted to compare the general flux results with experimental data collected from controlled virus infection experiments involving the three viruses studied here. Unfortunately, we did not find any studies that have directly measured metabolic flux changes during or after infection. There were, however, few datasets that considered changes in the cell medium or the serum upon infection, and we found notable overlaps with these data and predictions. For example, the model predictions for increased alanine exchange ('EX_ala(e)') in the DENV-optimized model were in line with the observed increase in alanine levels in the media of DENV2-infected versus -uninfected EA.hy926 cells [28]. Similarly, the upregulation of glycine, serine and threonine metabolic subprocesses (contained within the ‘amino acid’ subprocess in figure 3*a*) in the CHIKV- and DENV-optimized models matches with previous metabolomics studies of CHIKV- and DENV-infected human serum [29]. In the case of the ZIKV-infected model, we find that our prediction of the virus-induced upregulation of dihydrofolate reductase (DHFR) is in line with previous metabolomics studies of ZIKV-infected human serum [30].

We also collated gene expression data from several infection experiments, presenting expression levels before and after infection (see electronic supplementary material and file S9). Unfortunately, none of these studies was conducted on the modelled host, the human macrophage cell, but instead used other human cell lines, and as a result we did not find a strong overall correlation between expression changes in metabolic genes and model-based flux changes (in line with the previously observed lack of correlation between enzyme expression and metabolic flux changes [31]).

### 2.4. The integrated model highlights enforcement of host-optimal flux ranges as an antiviral strategy to suppress viral production

As the host-optimal and virus-optimal flux ranges within the integrated model differ, we hypothesize that the model can be constrained in a way to limit viral production (see *Methods*). To test and use this hypothesis, we use the integrated stoichiometric model to identify the host reactions, which, when constrained, limit virus production the most. This analysis can be implemented in different ways, for example through constraining of flux values to zero (i.e. reaction ‘knock-outs’). Applying such knock-outs, we find several reactions that limit virus optima, but all of these also result in significant reduction in host optima (electronic supplementary material, file S3). To identify if there are any reactions that can perturb virus production, while maintaining the host viability, we constrained reaction fluxes to ranges that are derived from the FVA described above. In particular, we identified flux ranges that still allowed for the attainment of the host-optimal state but were outside of the range allowed by the virus-optimal state (see *Methods*).

**Figure 4.**
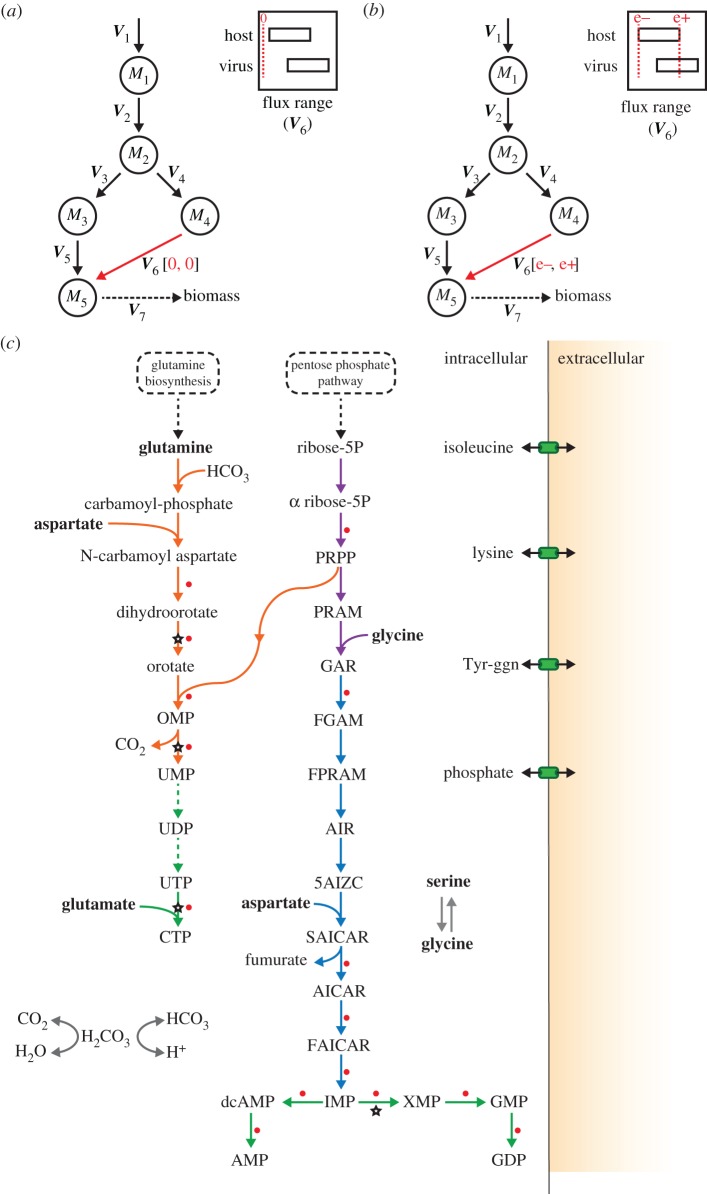
Prediction of antiviral targets [reactions] from single-reaction perturbations in a virus-optimized system for CHIKV, DENV and ZIKV. (*a*) Simplified schematic for single-reaction knockouts, where flux ranges for a desired reaction flux *v* are shown for both host- and virus-optimized systems. Square brackets, associated with specific flux vector indices 1–7, denote the lower (lb) and upper (up) flux bounds ([lb,ub]). Under knockout conditions both lb and ub are set to zero. (*b*) Simplified schematic for single-reaction enforcements, where flux ranges for a desired reaction flux *v* are shown for both host- and virus-optimized systems. Square brackets, associated with specific flux vector indices 1–7, denote the lower (lb) and upper (up) flux bounds ([lb,ub]). Under host-derived enforcement conditions, lb and ub are set to e− and e+, respectively (see *Methods*). (*c*) Reaction pathway schematic showing the top 29 reactions from host-derived flux enforcement analysis and their associated antiviral drugs and inhibitors. Key reactions inhibiting virus optima when flux ranges derived from host and virus flux variability analysis are enforced (the same reactions listed in electronic supplementary material, file S4). Abbreviations used for the compounds and reactions are as in electronic supplementary material, tables S2 and S3, respectively. Some of the identified reactions are interconnected, forming pathways. Coloured reaction arrows indicate pathways associated with subsystems: orange, pyrimidine synthesis; purple, pentose phosphate pathway; blue, purine synthesis; green, nucleotide biosynthesis. The starting metabolites into these pathways, glutamine and d-ribose 5-phosphate, are derived from glutamine biosynthesis and pentose phosphate pathways. Reactions targeted by a known antiviral or inhibitor are marked by white and red filled stars and circles, respectively. Complete list of antiviral compounds from which the matches were obtained is provided as electronic supplementary material, table S1. A complete list of inhibitors and the associated reactions is provided as electronic supplementary material, file S4*.* A complete list of enforcement results for all reactions is provided as electronic supplementary material, files S2 and S5.

This approach highlights a set of reactions that result in different levels of reductions in the virus optima of CHIKV, DENV or ZIKV, while not affecting the host optima (as expected from the way we set the flux constraints; see *Methods*). We identify 29 reactions that can reduce the virus optima to below a threshold [80%] of the original value for at least one virus (see electronic supplementary material, file S4; full results are provided in electronic supplementary material, file S3). Interestingly, many of these 29 reactions are interconnected and are involved in the *de novo* synthesis of RNA nucleotides (both purine and pyrimidine pathways) and amino acid interconversions (figure 4). Particular examples include reactions directly involved in the synthesis of adenosine, guanosine and uridine/cytidine nucleotides, and upstream reactions such as those involving inosine monophosphate (IMP) and orotidine monophosphate (OMP). We found that these predictions were robust towards changes in key model assumptions (see electronic supplementary material).

### 2.5. The predicted antivirals match known ones and include new host-based antiviral candidates

These identified reactions are potential antiviral targets, in the sense that altering their fluxes can limit virus production within the host. Thus, we explored if these reactions match with known antiviral drug targets. Performing a literature analysis, we found that there are currently 10 antivirals, specific to RNA viruses, and that these target only five unique metabolic enzymes (see electronic supplementary material, table S1). Of these five drug targets (and the associated drugs), one has been experimentally verified to be effective against CHIKV (inositol-5′-monophosphate dehydrogenase; *IMPD*) [32]; and another against DENV (dihydroorotate dehydrogenase; *DHORD9*) [33]. While the other three targets have been verified to be effective against some RNA viruses [34], they are yet to be tested against CHIKV, DENV and ZIKV.

We found that out of these five known antiviral targets, all are implicated in our analysis. The three known antiviral target reactions involving the genes *IMPD* [32]; *DHORD9* [33]; and orotidine-5′-phosphate decarboxylase (*OMPDC*) [34] are found to perturb virus optima for all viruses (figure 4). The antiviral target S-adenosylhomocysteine hydrolase (*AHC*) [34] is predicted to affect only CHIKV optima and only to a level higher than the 80% cut-off we used in the above analysis. We note that setting *AHC* reaction flux to zero abolishes virus growth for all three viruses (see electronic supplementary material, file S3). Finally, CTP synthase, which has been indicated to exhibit an effect on several RNA viruses [34], is included in the model as two reactions which perform the same reaction. These are defined as using either ammonia (mediated by *CTPS1*) or glutamine (mediated by *CTPS2*) as a nitrogen source [35] and therefore not highlighted in our initial flux enforcement analysis focusing on a single reaction. When we constrain both reactions associated with these two reactions simultaneously at host-derived flux ranges, a reduction in all virus optima is observed.

### 2.6. Existing drugs can target many of the predicted additional host reactions affecting virus production

Considering the computationally predicted potential of the additional reactions identified as antiviral targets, we have searched for these reactions in a database of known inhibitor-like compounds [36]. We found that 15 of these reactions already have known compounds and, in some cases, existing drugs, targeting their catalysing enzymes, identified from the DrugBank and BRENDA databases (figure 4, and full list in electronic supplementary material, file S4). These findings present experimentally testable predictions on host reactions, the disruption of which could limit virus production. It must be noted, however, that our computational analysis identifies flux enforcement based on differences in host- and virus-optimal states of the model, where ‘enforcement’ can mean either reduction or increase in a given flux. By contrast, most of the currently known molecules act as enzyme inhibitors [36] and would be expected to reduce metabolic fluxes. For the reactions highlighted in figure 4, we find that all reactions are downregulated (decreased flux) compared to the virus-optimal flux for that reaction.

## 3. Discussion

We present a computational approach that combines application of FBA and FVA with the development of integrated host–virus metabolic models. We show that this approach recovers the known metabolic antiviral targets within a human macrophage cell and predicts new potential targets. These predicted reactions fall primarily onto pathways involving nucleotides and amino acids that are differentially used by the host and virus. The results of this study are in line with an integrated perspective that views the virus as an additional metabolic burden on the host cells that could be met or avoided by tinkering of host metabolic fluxes. The observed overlap between predicted reactions and known antiviral drugs gives confidence to this integrated modelling approach and highlights its potential as a rapid prediction tool to guide experimental design. This can be especially useful in the case of new and emerging viruses for which limited clinical and experimental data may be available to inform drug target identification.

The integrated stoichiometric metabolic modelling approach focuses on metabolic changes as a driver of virus production and does not consider factors associated with virus–host cell recognition, viral entry, lipid envelope production and release [37]. In addition, this approach does not consider possible additional dynamical transcription processes during viral infections, such as sub-genomic particle generation [38]. These additional mechanisms relating to the virus infection and production can possibly be incorporated in future dynamical models. The current application of the linear optimization on stoichiometric models (i.e. FBA and FVA) strictly assumes that host metabolism is at steady state, and thus prohibits analysis of the dynamics of cellular physiology. Such dynamics could be taken into account to a certain extent by imposing different flux constraints, which could be derived from proximal experimental data [16], through the development of simplified metabolic temporal models [10,11], or by combining dynamics with linear optimization on stoichiometric models [39,40]. Additionally, the extent of the missing information, such as genes, enzymes or reactions, in genome-scale stoichiometric models creates limitations on how much of the metabolic processes can be covered [41].

Future efforts to improving model curation and standardization [42] would open up the possibility of extensive analysis of host–virus pairings from a metabolic stance. Such modelling efforts would immensely benefit from a collection of appropriate, relevant experiment datasets. In particular, experimental analysis of cellular metabolic fluxes, as well as the determination of cellular uptake rates and metabolite requirements, can allow direct evaluation of the model. The presented findings already suggest that integrated host–virus models can highlight metabolic changes in the host and predict principal host metabolic processes that are linked to host–virus compositional mismatches and that can be used to combat virus production without altering host functions. In particular, analysis of extended flux enforcement strategies such as flux limitations on double and triple reaction combinations might identify virus-specific drug combinations. Combining this with the future development of additional host–virus integrated models, covering many cell and virus types, can thus allow a fruitful route to the computational guiding of experimental antiviral drug discovery.

## 4. Methods

### 4.1. Flux balance analysis

FBA is a mathematical, constraint-based modelling method used to simulate reconstructions of cellular metabolic networks [43]. FBA assumes that the metabolic model is at steady state, and uses linear optimization to predict a set of fluxes that is compatible with this assumption and the enforced upper and lower flux bounds, and is optimal given a defined objective function. While different biologically relevant objective functions can be constructed, a commonly employed one involves a pseudoreaction representing cellular biomass production or maintenance. The linear optimization problem used in FBA can be generally formalized as follows:
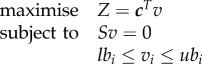
where *S* is the stoichiometric matrix, defining the stoichiometry of metabolites in different reactions, *v* is a vector of metabolic reaction fluxes, and ***c*** is a vector of the same size as *v* and encoding the objective function *Z* (e.g. *c* might be a binary vector with 1 at the index of the reaction(s) composing the desired objective, and 0 elsewhere). Additional constraints on each reaction flux *v_i_*, is defined through the minimal (*lb*) and maximal (*ub*) flux bounds.

Here, we implement FBA and its close variant FVA for specific analyses of an integrated host–virus metabolic model as described in further detail below. FVA provides an additional layer of information to FBA by predicting the permissible flux values for each reaction. Thus, FVA yields two flux distribution vectors, which predict the minimum and maximum flux value that a reaction can have (defined as its flux range) given the objective function.

### 4.2. Generation of virus biomass objective functions

To implement the FBA approach to studying virus infections from a metabolic stance, we define a pseudoreaction accounting for the production of virus particles from its constituents. We call this reaction a virus biomass objective function (VBOF). To account for metabolic fluxes associated with the virus production, the VBOF needs to capture the stoichiometry of nucleotide, amino acid and associated energy metabolites relating to virus production, similar to biomass production function used for microbial metabolic models [44]. We derive the metabolic stoichiometry of virus production from the viral genome sequence, the subsequently encoded proteins, the copy number of those proteins, and knowledge of the energetic requirements for peptide bonds and phosphodiester bonds. As previously mentioned, we do not include the virus envelope in the VBOF due to a lack of stoichiometric information regarding virus-associated lipids. We also do not include lipids due to the lack of dynamics in the model (therefore virus entry/exit is not modelled, where the exit is the location of virus envelope acquisition). Details of the VBOF derivation are given below, while a schematic of VBOF generation is included as electronic supplementary material, figure S1.

#### 4.2.1. Genome and protein information for the viruses

The genome sequences used in the present study are obtained from the NCBI genomic database [45] using the following accession numbers and accessed in March 2016; ZIKV: NC_012532.1, DENV: NC_001474.2, and CHIKV: NC_004162.2 (original files are provided on the Soyer group research website: http://osslab.lifesci.warwick.ac.uk/?pid=resources). Viruses can be classified by their replication methods, known as the Baltimore Classification System [46], and depending on this classification, a viral particle may contain more than a single copy of the genome. This is factored into the calculation of the nucleotide counts. In the presented study, all studied viruses fall into Group IV classification: they replicate their positive single-stranded RNA (+ssRNA) genome via a negative ssRNA (−ssRNA) intermediate. Therefore, the counts of the nucleotides in the negative strand is equal to the counts of the complementary nucleotide in the positive strand, i.e. count of A on (+/−) strand = count of U on (−/+) strand, and similarly for G and C counts. The count for each RNA nucleotide (adenosine (A), cytidine (C), guanine (G) and uracil (U)) can be taken directly from the virus genome sequence: RNA uses U in place of thymine (T); therefore, T must be replaced with U from the genome sequence read-out. In this study, all the viruses have two categories of polyproteins that compose the proteome: structural and non-structural. The amino acid sequence of these two polyproteins, and indeed any genome-derived protein sequences, are obtained from gene annotations of the viral genomes as provided in the NCBI genome entries (see above for NCBI entries used). The different subcategories of the viral proteome may be differentially incorporated into a single virus particle. For the viruses studied here, the structural and non-structural polyproteins are expressed in a ratio that is derived from the overall virus structure (i.e. proteins in the capsid or nucleocapsid) [18]. The ratio is 1 : 240 for CHIKV [18], and 1 : 180 for DENV/ZIKV [17]. More broadly, the ratio of different protein classes in a single virus particle can be derived from the overall virus structure or directly from literature/experimental evidence.

#### 4.2.2. Calculating nucleotide investment per virus

The total moles of each nucleotide in a mole of virus particle 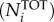 are obtained from their count in the virus genome 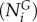 and replication intermediates 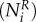, and multiplied by the genome copy number (*C*_g_):4.1
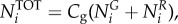
where the indexation is over nucleotides. The moles of nucleotides are then converted into grams of nucleotide per mole of virus (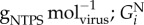), by multiplying 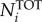
with the respective molar mass (g mol^−1^) of the nucleotides 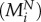:4.2
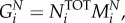
where the indexation is over nucleotides. Summing 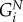 over all nucleotides and combining this with the similar calculation for amino acids allows us to get the total molar weight of the virus regarding nucleotides and amino acids (*M_v_*; see equation (4.15) below). Finally, the stoichiometric coefficients of each nucleotide in the VBOF are expressed as millimoles per gram of virus (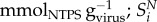):4.3
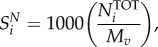
where the indexation is over nucleotides.

#### 4.2.3. Calculating amino acid investment per virus

The total moles of each amino acid per mole of virus particle 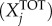 is obtained similarly using the sequence information of structural 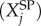 and non-structural 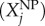 proteins. Counts of each amino acid in these proteins is multiplied by the respective copy numbers of these proteins (*C*_sp_ and *C*_np_):4.4
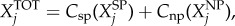
where the indexation is over amino acids. *C*_np_ is 1 for all viruses studied here, while *C*_sp_ is 240 for CHIKV [18], and 180 for DENV/ZIKV [17]. The moles of amino acids per mole of virus is then converted into grams of amino acid per mole of virus (
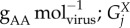
), by multiplying 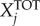 with the respective molar mass (g mol^−1^) of each amino acid (*M^X^*):4.5
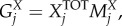
where the indexation is over amino acids. Finally, the stoichiometries of each amino acid in the VBOF is expressed as millimoles per gram of virus (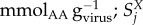):4.6
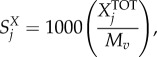
where the indexation is over amino acids.

#### Calculating ATP requirement for amino acid polymerization (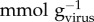)

4.2.4.

The polymerization of amino acid monomers requires approximately four ATP molecules per peptide bond [47], defined here as the constant *k*_ATP_ (=4) The overall moles of ATP (*A*^TOT^) required to form the structural (*A*^SP^) and non-structural (*A*^NP^) polyproteins are calculated from the respective amino acid counts:4.7
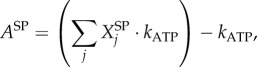
4.8
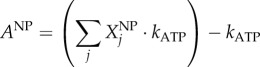
4.9
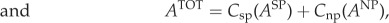
where the indexation is over amino acids. From *A*^TOT^, we calculate the stoichiometry of ATP in the VBOF as millimoles per gram of virus (*S*^ATP^):4.10
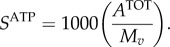


As ATP is hydrolysed in this process, the water requirement for polymerization 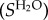 is equal to that of ATP. The products from the hydrolysis of ATP (ADP, P_i_ and H^+^) are also accounted for in the VBOF (see equation (4.16)).

#### Calculating pyrophosphate (PP_i_) liberation from nucleotide polymerization (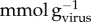)

4.2.5.

The polymerization of nucleotide monomers to form the RNA viral genome liberates a PP_i_ molecule [47], defined here as the constant *k*_PPi_ (=1). The overall moles of PP_i_ (*P*^TOT^) required to form the viral genome (*P^G^*) and replication intermediates (*P^R^*) are calculated from the respective nucleotide counts:4.11
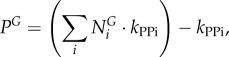
4.12
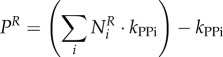
4.13
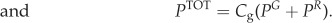


To convert this into the PP_i_ stoichiometry in the VBOF as millimoles per gram of virus (*S*^PPi^), we again use the overall molar mass (g mol^−1^) of one mole of virus:4.14
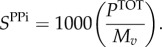


#### 4.2.6. Calculating total viral molar mass

The total molar mass of the virus *M_v_* is calculated from the total mass of the genome and proteome components as4.15
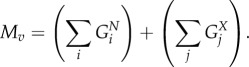


#### 4.2.7. Final construction of the VBOF

The left- and right-hand side terms of VBOF are based on the above calculations of stoichiometric coefficients. The final stoichiometry for the VBOF (pseudoreaction) is4.16
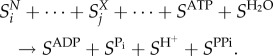


This pseudoreaction accounts for the virus' biomass, and the energy requirements associated with its production, and can be incorporated into stoichiometric metabolic models of the host to represent the presence of a virus in that system.

### 4.3. Construction of the human macrophage iAB-AMØ-1410 metabolic model

The human macrophage metabolic model, iAB-AMØ-1410, was constructed in a previous study [25]. This model was generated using clinical transcriptomic datasets, collected from variant patients’ alveolar macrophages, and using this to prune a set of reactions from a previous human genome-scale stoichiometric model of metabolism, RECON [48]. The objective function of iAB-AMØ-1410 was generated in line with previous protocols [44] using experimental and literature data to determine the biomass maintenance of a human macrophage cell per hour [25].

### 4.4. Integration of iAB-AMØ-1410 and Chikungunya, Dengue and Zika viruses

The VBOFs for the three viruses (CHIKV, DENV and ZIKV) were integrated into three separate instances of the ‘host’ macrophage model (iAB-AMØ-1410) (original files are provided as electronic supplementary material, file S7). In each case, the respective VBOF was appended into the existing macrophage model, with a lower flux bound of zero and an upper bound of infinity, reflecting no upper constraints on this flux [49]. No other metabolites or reactions were added to any of the models. All of the individual flux bounds of the model reactions were used as previously set [25], but any bounds set to −1000 or 1000 are replaced with infinity, because the use of infinity, rather than arbitrarily large values, is shown to be a more robust approach to represent unbounded reactions in a linear programming model [49]. We also confirmed that the use of arbitrary large bounds (such as −1000/1000) instead of infinity does not change the presented results qualitatively. A set of subprocesses, derived from known aggregate subsystems [50], were appended as metadata to each individual host–virus model and linked with the pre-existing defined subsystems. A full description of the subsystems and mapping of reactions into these are supplied in electronic supplementary material, file S3. The used integrated model is provided in a computer-readable (SBML) format with the publication.

### 4.5. Characterizing the stoichiometric differences between host and virus

For both the host (iAB-AMØ-1410) and viruses (CHIKV, DENV and ZIKV) we have pseudoreactions that capture the metabolic requirements for the maintenance/production of their respective biomass. By comparing these pseudoreaction stoichiometries, we can quantify the differences in amino acid and nucleotide requirements to fulfil the host or virus objectives. To do so, we calculate the fold change in nucleotide and amino acid usage by normalizing their individual stoichiometric coefficients against the sum of stoichiometries of all metabolites present in the objective function (other than ATP):4.17
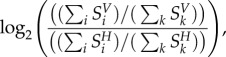
where indexation *i* is over nucleotides (or amino acids) and *k* is over all biomass precursors, and the subscripts *H* and *V* indicate the use of the host and virus biomass functions, respectively. A positive value indicates a higher usage of nucleotide (or amino acid) *i* by the virus than the host, while a negative value indicates a lower usage.

### 4.6. Comparison of host- and virus-optimized states

For all analyses, the generated host–virus integrated models were optimized, and reaction fluxes predicted using the linear optimization approach known as flux balance analysis (FBA) [43]. Linear optimization is a mathematical technique that optimizes a given function under a set of constraints defined by mathematical inequalities. In the context of metabolic models, the constraints correspond to limitations on reaction fluxes, while the function to be optimized can be defined as the flux in a specific reaction. While several biologically plausible objective functions can be defined [51], a standard approach is to define a pseudoreaction that describes biomass production from its constituent parts, and then optimize the flux to this reaction, as we have done here. As the set of constraints includes constraints on uptake reactions, this application of FBA results in the prediction of optimal biomass production flux for a specific uptake flux. In other words, FBA optimizes for biomass yield from given substrates assumed to be present in the media. In this work, we apply FBA to optimize a combined host–virus metabolic system to satisfy either the host or virus objective function (as described above) and study the resulting flux predictions.

To simulate a virus-optimal state, the models are optimized using the respective VBOFs of CHIKV, DENV and ZIKV viruses as the objective function, while to simulate a host-optimal state, the models are optimized using the existing biomass maintenance reaction for the human macrophage as presented in [25]. Besides running linear optimization to find the optimal flux sets under each scenario, we have also performed a flux variability analysis (FVA) [52], which provides flux ranges for each reaction that still would allow attainment of given host/virus optima. The FVA approach is shown to be more robust to instabilities associated with prediction and comparison of a single optimal flux sets [49]. For each reaction in the model we compared the resulting flux ranges from FVA under host and virus optimization, by evaluating the mean value of the allowed flux range for each reaction (*A_i_*) and then collating the mean flux values for reactions associated with given subprocesses (aggregated subsystems) as a percentage of total flux through that process. More formally, we define the differential distribution of reaction flux for each subprocess (*i*) between the host- and virus-optimized models in terms of a fold change:4.18
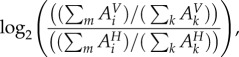
where the indexation *k* is over all reactions of the model, while the indexation *m* is over reactions that belong to subprocess *i*. The superscript indicates the use of flux values from the host (*H*)- and virus (*V*)-optimized models, respectively. A positive value indicates a higher mean flux for subprocess *i* in the virus- versus host-optimized model, while a negative value indicates a lower mean flux.

### 4.7. Reaction knockout and host-derived flux analyses

To find reactions that can preferentially alter virus-optimized state of the model, we considered the effect of systematically constraining individual reactions. *Knockout analysis**.*** Knockout analysis considers the effect of systematically setting individual reaction fluxes to zero, and then attempting to maximize for VBOF. The knockout optima for the virus production reaction flux 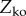 is then compared to the original flux over this reaction; 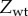. *Host-derived enforcement.* Host-derived enforcement considers the effect of maintaining a metabolic system in a host-optimized state while attempting to optimize the model for VBOF. For this approach, we systematically set individual lower and upper flux bounds of individual reactions to a specific flux range. For each reaction, this range (*ɛ^r^*) is derived from the corresponding minimum (*F^−^*) and maximum (*F^+^*) flux values for that reaction obtained from the FVA using the host (*H*) and virus (*V*) optimization (as described above). The range (*ɛ^r^*) is bounded by minimum (*ɛ^−^*) and maximum (*ɛ^+^*) flux values, which are given by the following conditional arguments:4.19
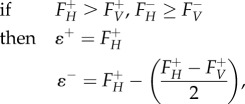
4.20
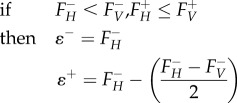
4.21
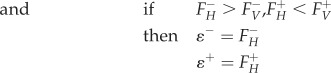


These calculated flux ranges for each individual reaction are then used to constrain the model, and the model is optimized for the VBOF. The resulting optima for the virus production reaction flux, 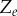 is recorded and compared to the original optimal value, 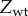.

## Data accessibility

All analyses are performed using an author-developed package written in Python; the ViraNet. This analysis tool is available under an academic, non-commercial use licence on author webpages at http://osslab.lifesci.warwick.ac.uk/?pid=resources.
